# Reflexive priority of biological motion in attentional orienting: evidence from conflict resistance in complex visual settings

**DOI:** 10.1186/s41235-026-00721-1

**Published:** 2026-03-22

**Authors:** Xinyi Huang, Shujia Zhang, Li Wang, Yi Jiang

**Affiliations:** 1 State Key Laboratory of Cognitive Science and Mental Health, Institute of Psychology, Chinese Academy of Sciences, Beijing, 100101 China; 2 Department of Psychology, University of Chinese Academy of Sciences, Beijing, 101408 China

**Keywords:** Biological motion, Social attention, Eye gaze, Reflexive, Conflict

## Abstract

Humans involuntarily orient their attention to walking direction of biological motion (BM), a crucial skill for adaptive survival and social interaction. While previous studies have been limited to isolated BM displays, real-world scenarios typically include BM alongside multiple competing stimuli, hampering the translation of laboratory insights into practical applications. Here, we introduced simultaneously presented BM cues and other social (eye gaze) or nonsocial (arrow) cues into a modified central cueing paradigm, reassessing the reflexive nature of BM-induced attention from the perspective of conflict resistance. Results showed that the attentional orienting elicited by BM was robust enough to resist interference from peripheral arrows throughout the task yet interfered with central arrow processing. This unique asymmetric interference effect highlights the reflexive priority of BM over nonsocial cues. Additionally, mutual interference between BM and eye gaze suggests that different types of social cues trigger attentional shifts with a considerable degree of reflexivity. Based on an interference-resilient criterion, these findings together imply that social attention is supported by a specialized mechanism shared across various social but not nonsocial cues. This mechanism potentially enables us to instinctively prioritize and orient toward social signals amid competing nonsocial cues in complex real-world settings, with direct implications for designing signaling systems in safety–critical contexts and developing early diagnostic tools for sociocognitive disorders such as autism.

## Significance statement

Humans possess an innate ability to direct their attention to walking direction of biological motion (BM), which plays an essential role in adaptive functioning and interpersonal interaction. Traditional studies concerning such fundamental capacity have relied on simplified paradigms with isolated cues, neglecting the fact that real life is crowded with multiple directional cues where efficient attentional orienting can be a matter of safety. The current study uncovers, for the first time, that this attentional orienting triggered by BM is reflexive enough to withstand conflict from concurrently presented nonsocial cues (i.e., arrow) and even to progressively affect their processing. Together with the rapid interference observed between social cues (i.e., BM and eye gaze), these findings shed light on a shared mechanism specialized for social but not nonsocial attention, broadening the understanding of how humans instinctively prioritize social signals in the complicated real world. Beyond theoretical insights, this work is highly relevant for advancing designs of attentional guidance systems in human–computer interface or safety-critical settings, and offers ecologically valid approaches to early autism diagnosis.

## Introduction

As social beings in the bustling world, we instinctively shift our attention toward the walking direction of those passing by. This automatic orientation to walking direction of biological motion (BM) likely evolved because it conveys critical information about others’ intentions, which is indispensable for human survival (e.g., timely responses to social opportunities or potential threats) and interpersonal interactions (Blake & Shiffrar, 2007; Wang et al., 2020). It is well documented that we possess a remarkable ability to effortlessly and precisely discern walking direction, even when BM cues are presented as only a dozen point-light dots attached to the head and major joints of the body (Johansson, 1973; Troje, 2002) and embedded in dynamic visual noise (Hirai et al., 2011; Troje & Westhoff, 2006) or presented in peripheral vision (Thompson et al., 2007). Crucially, this heightened sensitivity to walking direction of BM extends beyond mere perception and further affects behavioral responses. Previous studies have consistently demonstrated that walking direction of point-light BM can trigger reflexive attentional orienting (Liu et al., 2021; Shi et al., 2010; Wang et al., 2020; Yu et al., 2020). Specifically, despite being explicitly informed that walking direction is nonpredictive of the upcoming target location, observers still orient their visuospatial attention accordingly, leading to faster responses to targets appearing on the side signaled by BM cues. Such attentional orienting can be induced by local BM signals lacking global configuration, including cases where only the motion of the feet is displayed and observers are unaware of its biological nature (Wang et al., 2014). Interestingly, BM-induced attentional orienting behavior emerges early in life, observed in preschool children (Zhao et al., 2014) and infants as young as 3–6 months old (Bardi et al., 2015; Lunghi et al., 2019, 2020; Richards, 2000). In brief, these behavioral and developmental studies suggest that walking direction of BM is similar to eye gaze, a widely recognized social signal. Both serve as powerful central directional cues capable of reflexively orienting our attention toward others’ focus to facilitate social interaction and coordinate adaptive behavior (Driver et al., 1999; Friesen & Kingstone, 1998, 2003; Ristic et al., 2007; Yuan et al., 2023), a phenomenon known as social attention (Birmingham & Kingstone, 2009; Frischen et al., 2007).

Previous studies concerning attentional orienting induced by walking direction of BM have primarily employed single BM as central cues (Bardi et al., 2015; Liu et al., 2021; Lunghi et al., 2020; Shi et al., 2010). However, throughout daily life, we are commonly exposed to complex scenes bombarded with a multitude of stimuli, which can lead to conflicts between BM and other directional cues. For instance, when wayfinding in crowded transportation hubs (e.g., airports or train stations), we often find ourselves in a situation where the flow of pedestrians, the divergent glances of others, and numerous signage all vie for our attention. Such conflicts of multiple cues become particularly critical in high-speed driving, incident response, emergency rescue, or chaotic situations (e.g., panic or rioting), where distraction or misdirected attention could lead to severe, even life-to-death outcomes (Hinton et al., 2024; Sundfør et al., 2019; van Harten et al., 2021). Emergency evacuations exemplify such scenarios: we must rapidly reconcile conflicting directional information when some individuals move along one path while arrow signs indicate alternative routes. This real-world complexity necessitates a reassessment of the reflexivity of BM-induced attentional orienting observed in laboratories with isolated-cue displays: Is such attentional orienting reflexive enough to resist interference from conflicting directional cues? Moreover, a defining criterion for an automatic process is its resistance to interference from other processes while retaining the capacity to interfere with them (Besner et al., 2021b), raising the question of whether BM stimuli can modulate attentional shifts toward other cues. In sum, deciphering how we respond to BM signals in conflict situations could not only contribute to better measurement of the reflexivity of social attention in more real-world settings, but also directly inform practical applications in safety–critical signaling, navigation design, and human–computer interaction where rapid attentional orienting amid visual clutter is essential.

Nevertheless, to our knowledge, no studies to date have addressed the attentional orienting induced by simultaneously perceived and conflicting BM signals alongside other cues. Broadening the scope of cue types, only a few studies explored attentional orienting when eye gaze and arrow cues were presented concurrently (Besner et al., 2021a; Nummenmaa & Hietanen, 2009) and provided mixed evidence. In the dual-cue paradigm adopted by these studies, both cues were presented simultaneously with 50% validity (nonpredictive), and interference effects were defined as the reduction or elimination of attentional effect triggered by the central cue when it was paired with a simultaneous, oppositely oriented cue (e.g., in the periphery). One study (Nummenmaa & Hietanen, 2009) reported mutual and comparable interference effects between these two cues, while another study (Besner et al., 2021a) showed that arrow cues were resistant to interference from eye gaze. However, these findings diverge from those observed in isolated-cue conditions, where gaze elicits stronger reflexive attentional orienting compared to arrows (Friesen et al., 2004; Liu et al., 2021; Ristic et al., 2007). Such a discrepancy may arise from the use of arrow cues in a single schematic face or a face-like circle, potentially disrupting the intact perception of eye gaze (Fan et al., 2018). Additionally, the perceptual similarity between gaze and arrow cues in their directional information transmission (Gibson & Kingstone, 2006) brings inherent challenges for distinguishing their respective attentional mechanisms. It is thus important to introduce BM, a distinctively different yet potent social cue, into conflict settings to enable an indirect comparison of eye gaze and arrow cues through their respective interference effects on BM. Furthermore, the simultaneous presentation of BM along with these two typical cues (i.e., arrow, eye gaze) could offer a novel perspective on whether BM-induced social attention relies on specialized mechanisms that are functionally distinct from those engaged by nonsocial attention, and whether it shares common mechanisms with gaze-induced social attention. This may help clarify the long-standing debate regarding the distinctiveness of social attention (Callejas et al., 2014; Friesen et al., 2004; Lockhofen et al., 2014; Singh et al., 2022; Tipples, 2008; Uono et al., 2014; Wang et al., 2020), which highly relevant to interface design and signaling applications.

Therefore, the present study aimed to further probe into the attentional processing mechanisms when BM cues were presented centrally or peripherally and conflicting with nonsocial cues (arrows: Experiments 1–3 and 6) and other social cues (eye gaze: Experiments 4–5). Based on a modified central cueing task (Friesen & Kingstone, 1998) which has been widely employed to measure attentional effects (Joseph et al., 2015; Liu et al., 2021; Shi et al., 2010), participants were instructed to localize horizontally presented targets. These targets were preceded by combined yet independent cues, which are both nonpredictive regarding target location. Additionally, this study incorporates more trials per condition than previous research (Lockhofen et al., 2014; Nummenmaa & Hietanen, 2009; Shi et al., 2010; Wang et al., 2020) to further explore the temporal dynamics of processing conflicting stimuli over trials.

## Method

### Participants

A total of 120 graduate and undergraduate students aged 18 to 28 years (*M* ± *SD* = 23.09 ± 2.64) took part in the study. Each experiment included 20 participants (12 females in each of Experiments 1 to 5 and 11 females in Experiment 6). The sample size was determined by a prior power analysis utilizing G*Power 3.1.9.7 (Faul et al., 2007) for a within-subject analysis of variance (ANOVA), which indicated that 18 participants would afford 80% power for a 2 × 2 interaction with a medium effect size (*η*_*p*_^2^ = 0.08; Besner et al., 2021a). All participants had normal or corrected-to-normal vision and were naïve to the purpose of the experiments. They all gave written, informed consent before the experiment.

### Apparatus and stimuli

Stimuli were generated and displayed using MATLAB (Mathworks, Inc.) together with the Psychophysics Toolbox extensions (Brainard, 1997; Pelli, 1997) on a 19-in. CRT monitor (1280 × 1024 at 60 Hz). All stimuli were presented on a gray background (52.4 cd/m^2^) at a viewing distance of approximately 57 cm.

BM stimuli for all experiments were adopted from Vanrie and Verfaillie (2004), which were created by capturing the motion of a walking actor. These 3D motion capture coordinates were further rotated in depth and projected onto a 2D plane for display. Each BM sequence consisted of 13 white (108.5 cd/m^2^) point-light dots depicting the motions of the head and major joints (shoulders, elbows, wrists, hips, knees, and ankles), walking either leftward or rightward without any overall translational motion. Such a format systematically eliminated contour, texture, and depth information to isolate pure kinematic information from moving joints (Troje, 2002; Vanrie & Verfaillie, 2004). In line with the original recording (Vanrie & Verfaillie, 2004) and standard practice (Wang et al., 2020, 2024), a complete gait cycle was 1 s and contained 30 frames (33.3 ms/frame). The initial frame of BM stimuli was randomized for each trial to avoid participants’ prediction. In Experiments 1, 2, 3, and 6, a two-tailed point-light arrow was employed to better match the physical characteristics of the BM (e.g., dots’ number, size, and brightness). In Experiments 4 and 5, a neutral face image with gaze averted to the left or right was adapted from Ekman and Friesen’s facial expression image library (Ekman & Friesen, 1976). The image was cropped to eliminate features outside of the face (e.g., ears and hair). Moreover, the position of irises and pupils of the eyes was shifted to the canthi using Adobe Photoshop software, creating distinct leftward and rightward gaze cues (see Fig. 2). For each gaze direction (left or right), the angular deviation of the gaze from the center was kept consistent across trials.

### Design and procedure

#### Experiment 1

The experiment used a 2 (cue validity: valid vs. invalid) × 2 (direction congruency: congruent vs. incongruent) within-subject design. Cue validity was defined by whether the central cue indicated the same (valid) or opposite (invalid) side as the target appeared, while direction congruency referred to whether the direction of central cues was aligned with (congruent) or opposite to (incongruent) the direction of peripheral cues.

**Fig. 1:**
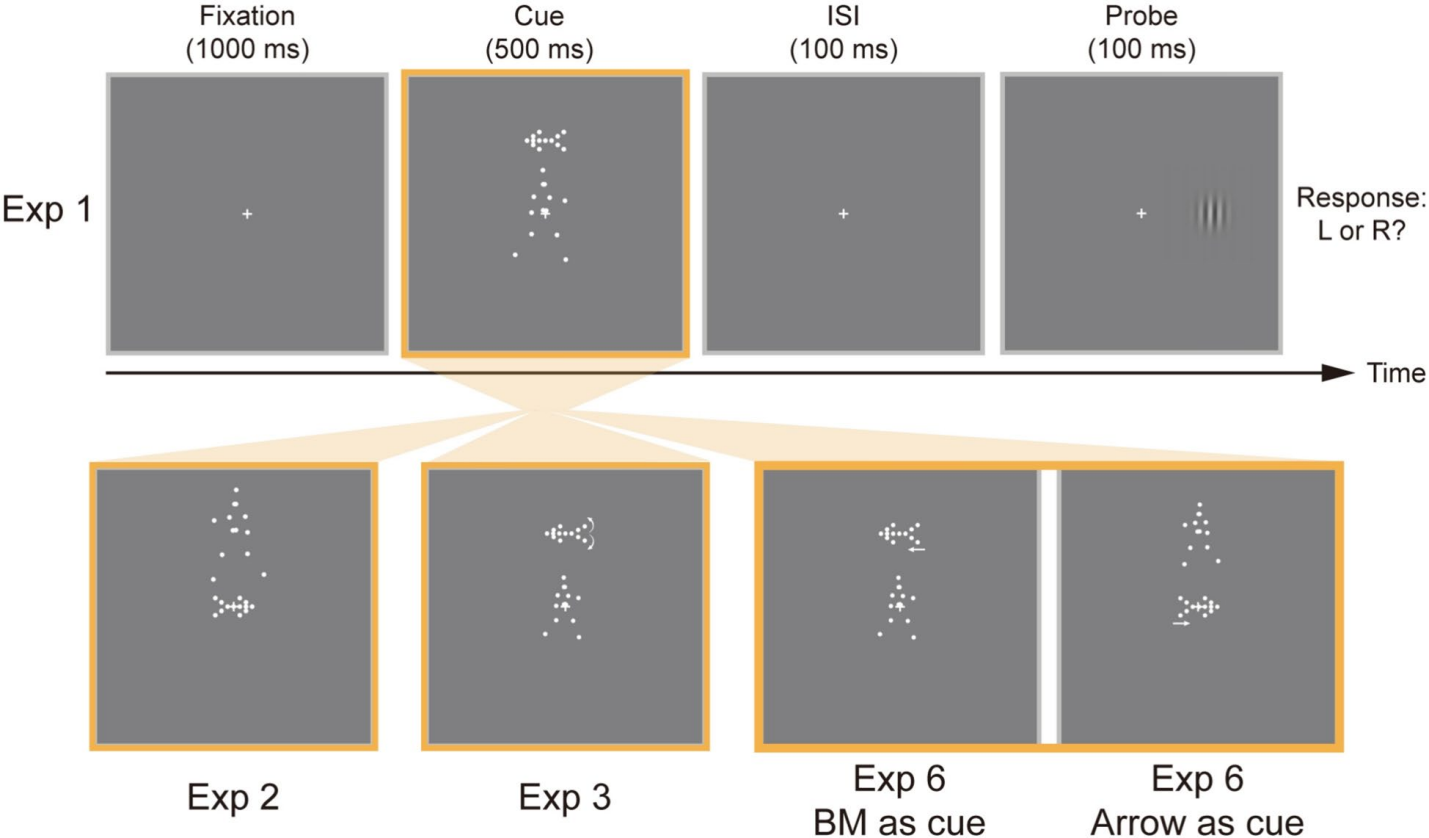
Schematic diagram of a single trial in Experiments 1, 2, 3, and 6. In Experiment 1, each trial began with a fixation lasting 1000 ms, followed by the simultaneous presentation of a central BM cue and a peripheral arrow cue (500 ms). After a 100-ms interstimulus interval (ISI), a small Gabor patch was displayed briefly (100 ms) as a probe on the left or right side of the fixation. Participants were required to rapidly and accurately press one of two arrow keys to indicate the probe location. The procedures of Experiments 2, 3, and 6 were similar to those of Experiment 1 except for different central and peripheral cues, with Experiment 6 additionally adopting a within-subjects design

Each trial began with fixation on a central cross (0.5° × 0.5°) within a frame (17.0° × 17.0°) that extended beyond the outer edge of the stimuli. After 1,000 ms, a point-light BM cue (3.2° × 5.7°) was superimposed on the central cross, together with a two-tailed arrow cue (2.4° × 1.3°) presented vertically 4.3° above or below the central cross. All stimuli were displayed simultaneously for 500 ms. After a 100-ms interstimulus interval (ISI), a small Gabor patch (1.1° × 1.1°) was briefly (100 ms) presented as a target on the left or right side of the fixation, 4.2° from the central cross. Participants were asked to press one of two arrow keys on a standard keyboard to indicate target location (left arrow key for left target and vice versa), as quickly as possible without compromising accuracy (see Fig. 1).

Throughout the whole task, participants were required to fixate on the central cross and explicitly informed that the direction of stimuli (central and peripheral cues) did not predict target location. There were 320 trials in total, divided into 4 blocks. Each block contained 80 trials and all conditions were counterbalanced, with 20 trials for each condition. Under each condition, the peripheral arrow cue had an equal chance of appearing above or below the central cross. Short rest breaks were provided after every 40 trials. Test trials were presented in a randomized order for each participant and conducted after 16 practice trials.

#### Experiment 2

Experiment 2 followed the same design and procedure as in Experiment 1, except that the arrow stimulus was employed as a central cue while the BM stimulus as a peripheral cue.

#### Experiment 3

Experiment 3 was similar in procedure to Experiment 1 but incorporated specific adjustments to better align BM and arrow stimuli. In particular, the size of central BM cues was appropriately reduced (2.2° × 3.9° instead of 3.2° × 5.7°). Meanwhile, the peripheral arrow cue was made dynamic, with both the angle of arrowhead and arrowtail expanding uniformly from 50° to 70° within 500 ms (ultimately consistent with the static arrows in Experiments 1 and 2).

#### Experiment 4

**Fig. 2:**
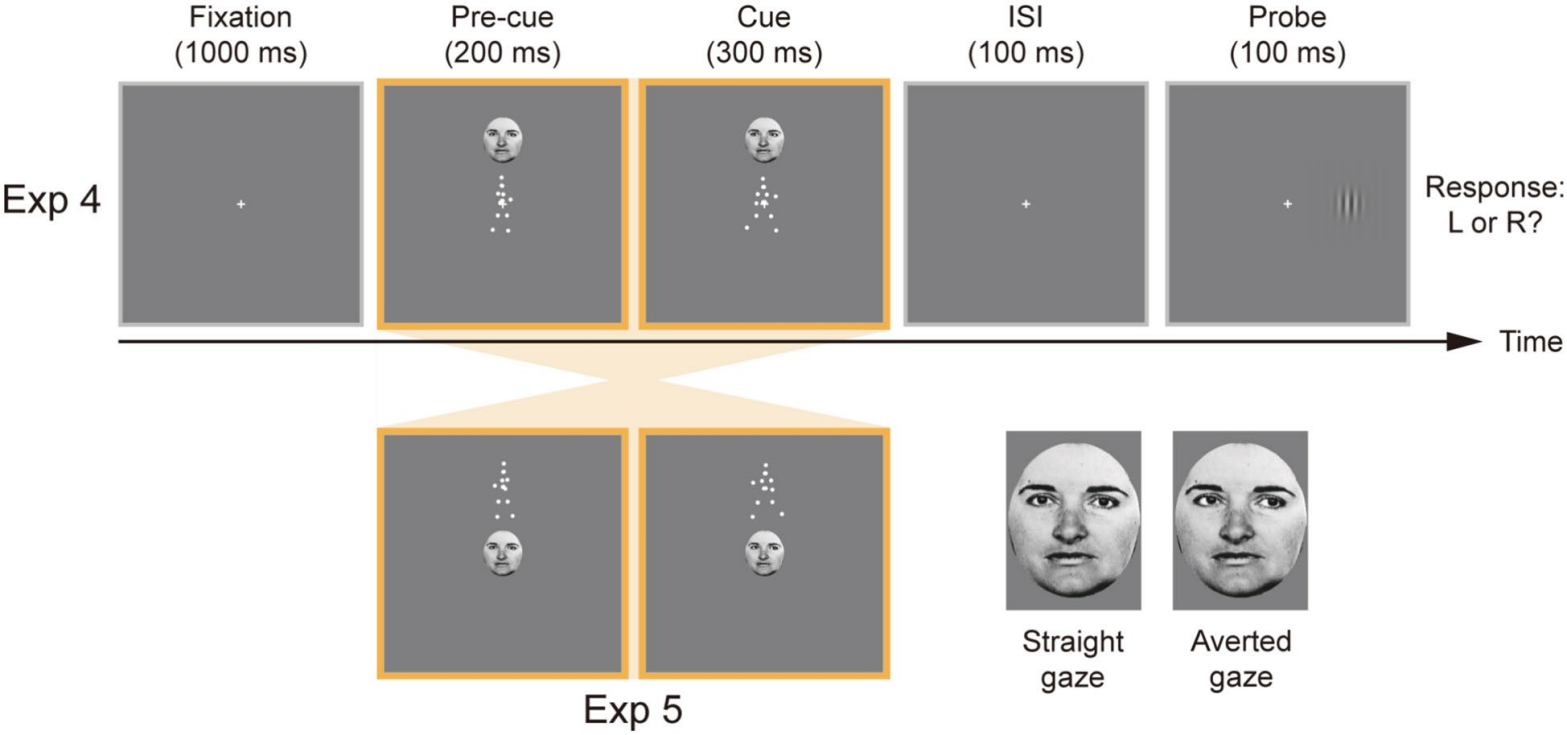
Schematic diagram of a single trial in Experiments 4–5. Experiment 4 followed the design of Experiment 1, with the key difference that eye gaze cues served as peripheral cues. Specifically, during the 500-ms BM display, a neutral face with straight gaze appeared briefly (200 ms) above or below the central cross, followed by a 300-ms gaze shift to the left or right. Experiment 5 reversed the positions of BM and eye gaze stimuli, while other procedures remained consistent with those in Experiment 4. Enlarged face stimuli depicting the straight gaze and averted gaze conditions are shown on the right

Contrary to Experiment 3, peripheral arrow cues were replaced by face images in Experiment 4 (see Fig. 2). Within the 500-ms BM presentation, a face with straight gaze (2.4° × 3.1°) was displayed vertically above or below the central cross at a distance of 4.3° for 200 ms, followed by a 300-ms gaze cue (the same face but with leftward or rightward gaze).

#### Experiment 5

Experiment 5 was identical to Experiment 4 except that BM and eye gaze stimuli switched their roles, namely eye gaze stimuli were used as central cues and BM stimuli were used as peripheral cues.

#### Experiment 6

Experiment 6 followed a similar procedure to Experiments 1–5 but adopted a within-subjects design and incorporated a new dynamic arrow stimulus, which allowed for more robust comparison of social versus nonsocial attention within a dual-cue conflict paradigm. In particular, this experiment was divided into two sessions focusing on specific central cues (BM, arrow). In the BM-cue session, central BM cue (2.2° × 3.9°, identical to the size used in Experiments 3–5) appeared for 500 ms while peripheral arrow cue initially appeared at 4.3° visual angle vertically above or below the central cross for 200 ms. The arrow subsequently moved 0.06° in its indicated direction (i.e., leftward for a leftward cue, rightward for a rightward cue)—equivalent to the displacement of irises and pupils during gaze shifts in Experiments 4–5—and held this position for the remaining 300 ms. In the arrow-cue session, arrows served as central cues and BM as peripheral cues, with all other procedures unchanged. Such modification made arrows more comparable to BM and eye gaze stimuli while enhancing their directional salience and dynamic properties. Each session contained 320 trials, consistent with the design in Experiments 1–5. The order of the two sessions was counterbalanced across participants. Before the experiment, participants performed 16 practice trials, with central and peripheral cues identical to those used in the subsequent first session.

### Data analysis

To evaluate the reflexivity of attentional orienting induced by central cues against peripheral cues, we extracted the response time (RT) in each trial for each participant. Following established practices in social attention research (Nummenmaa & Hietanen, 2009; Yuan et al., 2023), trials with incorrect responses, anticipations (RTs shorter than 100 ms), and retardations (RTs longer than 1000 ms) were filtered beforehand, followed by the removal of outlier trials with RTs beyond 2 standard deviations (*SD*) from each participant’s mean value. Overall, 6.07% of trials were excluded from analysis (Experiment 1: 6.98%, Experiment 2: 5.89%, Experiment 3: 5.95%, Experiment 4: 4.86%, Experiment 5: 6.05%, Experiment 6: 6.38%). Mean RTs were entered into a 2 × 2 repeated measures ANOVA with two within-subject factors of cue validity (valid vs. invalid) and direction congruency (congruent vs. incongruent). A significant interaction effect would suggest that attentional orienting induced by central cues is modulated by conflicting peripheral cues. Specifically, attenuated attentional orienting in the incongruent relative to congruent condition would indicate an interference effect. Furthermore, to directly compare attentional orienting with and without conflict while minimizing individual differences in response speed (Wang & Theeuwes, 2020; Wang et al., 2024; Zhang et al., 2025), normalized cueing effects (i.e., RT difference between the valid and invalid conditions divided by their sum, namely (RT_invalid _− RT_valid_)/(RT_invalid_ + RT_valid_); see also Gomez et al., 2018; Ji et al., 2020; Wang & Theeuwes, 2020; Yu et al., 2023) were calculated for each direction congruency condition (congruent, incongruent). Paired *t* tests comparing normalized cueing effects between the two conditions provided further evidence for the reflexivity of attentional orienting induced by central cues in conflict.

**Fig. 3:**
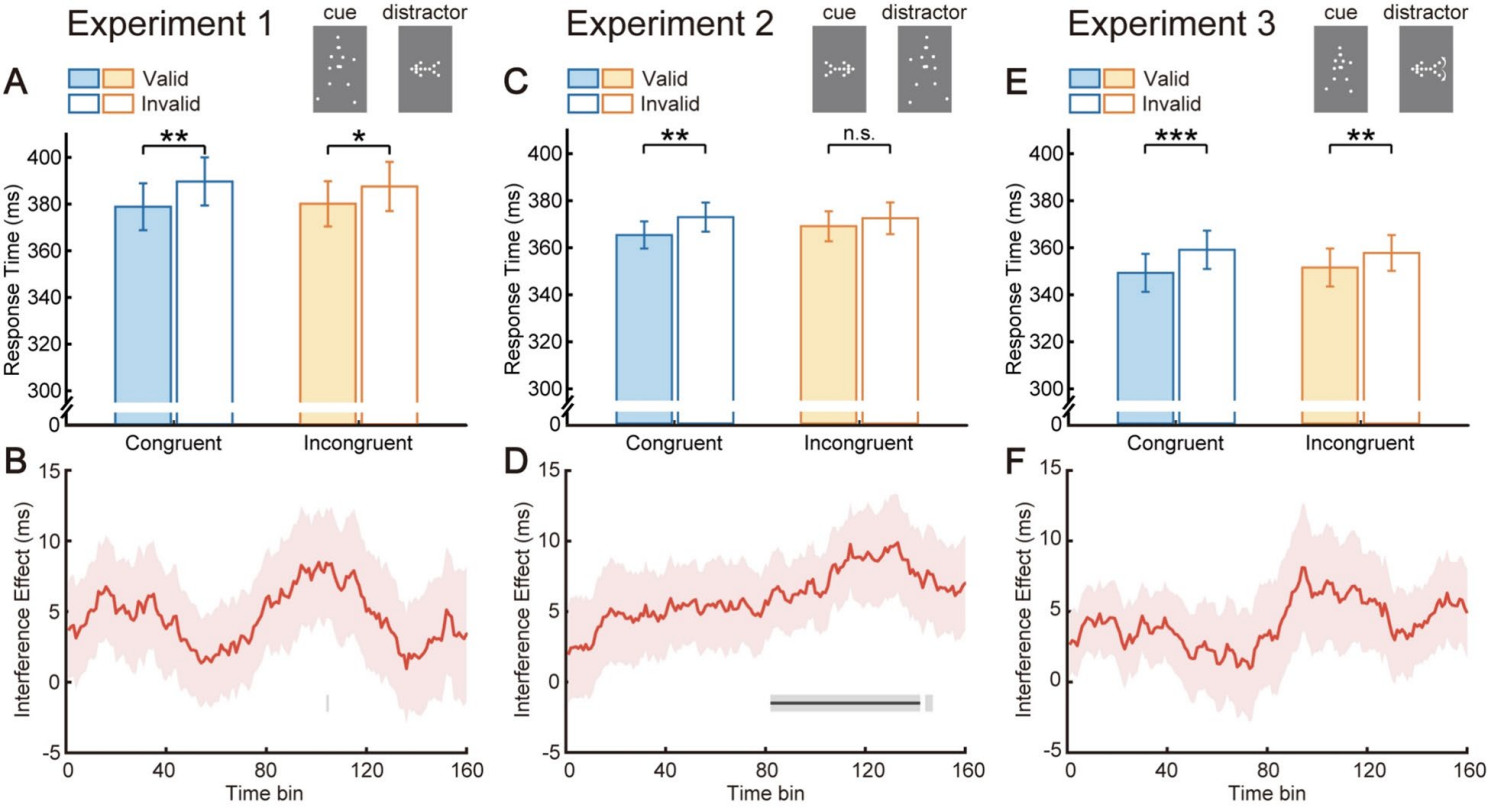
Results from Experiments 1–3. In Experiment 1, central BM cues stably induced a reflexive attentional cueing effect even when conflicting with a peripheral arrow pointing in the opposite direction (**A**, **B**). Such resilience to conflict vanished in Experiment 2 when arrows served as central cues and BM served as peripheral cues (**C**, **D**). After better matching these two stimuli, Experiment 3 replicated results observed in Experiment 1 (**E**, **F**). *p < 0.05; **p < 0.01; ***p < 0.001; n.s., not significant. In the panels below (**B**, **D**, **F**), red shaded areas represent standard errors; horizontal gray lines indicate time bins with significant interference effects at *p* < 0.05, while black lines indicate significant time bins after cluster-based permutation correction

Given that attention would fluctuate in the experiment, thereby averaging RTs data across all trials would obscure these dynamics and yield coarse estimates (Esterman et al., 2013). Here, we innovatively utilized a sliding window method to precisely portray how the resistance of attentional orienting to peripheral interference varies over time. The interference effect was defined as the difference between cueing effects in congruent and incongruent conditions ((RT_invalid_congruent _− RT_valid_congruent_) − (RT_invalid_incongruent _− RT_valid_incongruent_)). Its temporal trend was then assessed across trials. Specifically, 160 trials were set as a time bin, moving over trials with the step size of one trial. For instance, bin 1 included trials from the 1st to the 160th, bin 2 from the 2nd to the 161st and so forth, with bin 161 covering trials from the 161st to the 320th, thereby encompassing all trials. The running average of the interference effect was calculated from RTs under the four conditions of the 160 trials in each time bin, as drawn in Fig. 3A. This bin length ensured reliable estimates of interference effects by providing approximately 40 trials per condition within each time bin, comparable to prior research (Liu et al., 2021). Subsequently, consecutive one-sample *t* tests assessed interference effects across all time bins, followed by a cluster-based permutation test to avoid potential problems arising from multiple comparisons (Groppe et al., 2011). Clusters were formed when neighboring time bins had *t*-values exceeding a threshold (*p* < 0.05). Cluster mass, derived from the sum of *t*-values within each cluster, was compared against a null distribution generated from 2000 random permutations. When cluster mass fell beyond 95% of this distribution (*α* = 0.05), interference effects in time bins of the cluster were deemed statistically significant. Finally, to more explicitly illustrate the temporal trends, all time bins were further divided into two parts, the early and late halves (80 bins per section, with bin 161 removed), and one-sample *t *tests were conducted on individual data for mean interference effects in each period.

## Results

### Experiment 1

In Experiment 1, the repeated measures ANOVA showed a main effect of cue validity (*F*(1, 19) = 12.58, *p* = 0.002, *η*_*p*_^2^ = 0.40), indicating that even when explicitly informed that stimuli direction was not predictive of the target location, participants responded significantly faster to targets appearing on the same side of walking direction of BM cues (valid condition, *M* ± *SE* = 379.52 ± 9.84 ms) than on the opposite side (invalid condition, *M* ± *SE* = 388.64 ± 10.34 ms). Such a result confirms that central BM cues can effectively induce attentional orienting, as expected and consistent with previous studies where BM cues were presented in isolation (Shi et al., 2010; L. Wang et al., 2020; R. Wang et al., 2024). Importantly, there was no significant main effect of direction congruency (*F*(1, 19) = 0.07, *p* = 0.793, *η*_*p*_^2^ < 0.01) nor interaction between these two factors (*F*(1, 19) = 1.16, *p* = 0.295, *η*_*p*_^2^ = 0.06; see Fig. 3A). Normalized cueing effects further corroborated these outcomes, which again revealed no significant difference between the congruent and incongruent conditions (0.014 vs. 0.009, *t*(19) = 1.11, *p* = 0.279, Cohen’s *d* = 0.25, 95% CI for the mean difference [− 0.004, 0.014]). Collectively, these results jointly demonstrated that the direction of peripheral arrows could not affect the reflexive attentional orienting induced by central BM cues.

Notably, the temporal analysis using a sliding window method revealed that, no time bins exhibited significant interference effects after the cluster-based permutation test (see Fig. 3B). Moreover, the mean interference effect was neither significantly different from 0 for the early period (*M* ± *SE* = 3.99 ± 3.30 ms, *t*(19) = 1.21, *p* = 0.241, Cohen’s *d* = 0.27, 95% CI for the mean difference [− 2.90, 10.89]), nor for the late period (*M* ± *SE* = 5.15 ± 3.93 ms, *t*(19) = 1.31, *p* = 0.206, Cohen’s *d* = 0.29, 95% CI for the mean difference [− 3.08, 13.37]). Therefore, it is further confirmed that the reflexive attentional orienting induced by walking direction of BM was stably resistant to interference from peripheral arrows throughout the whole task, which may result from higher automation and reflexivity of cueing effects evoked by social cues than nonsocial cues (Friesen & Kingstone, 1998; Friesen et al., 2004; Langton & Bruce, 1999).

### Experiment 2

In Experiment 2, we further investigated whether peripherally presented BM stimuli could affect the attentional orienting induced by central arrow cues. Results revealed a significant main effect of cue validity (*F*(1, 19) = 7.57, *p* = 0.013, *η*_*p*_^2^ = 0.29), but no main effect of direction congruency (*F*(1, 19) = 1.15, *p* = 0.296, *η*_*p*_^2^ = 0.06). Crucially, the interaction between cue validity and direction congruency was marginally significant (*F*(1, 19) = 4.37, *p* = 0.050, *η*_*p*_^2^ = 0.19), suggesting potential interference of peripheral BM on arrow-induced attention. Post hoc *t* tests found a significant attentional orienting effect triggered by central arrow cues in the congruent condition (365.40 ms vs. 373.00 ms, *t*(19) = 3.14, *p* = 0.005, Cohen’s *d* = 0.70, 95% CI for the mean difference [2.53, 12.66]). However, this orienting effect disappeared in the incongruent condition (369.12 ms vs. 372.51 ms, *t*(19) = 1.67, *p* = 0.112, Cohen’s *d* = 0.37, 95% CI for the mean difference [− 0.87, 7.65]; see Fig. 3C). In correspondence with the result, the normalized cueing effect decreased in the incongruent condition compared to the congruent condition (0.010 ± 0.003 vs. 0.004 ± 0.003; *t*(19) = 2.03, *p* = 0.057, Cohen’s *d* = 0.45, 95% CI for the mean difference [− 0.0002, 0.012]). Overall, these findings together revealed that peripheral BM cues had a tendency to interfere with the cueing effect induced by central arrow cues.

Additionally, significant interference effects were discovered in bin 82–142 (see Fig. 3D). One-sample *t* tests similarly found that the mean interference effect in the late period (bin 81–160) was significant (early period: *M* ± *SE* = 4.69 ± 2.84 ms, *t*(19) = 1.65, *p* = 0.116, Cohen’s *d* = 0.37, 95% CI for the mean difference [− 1.27, 10.64]; late period: *M* ± *SE* = 7.54 ± 2.89 ms, *t*(19) = 2.61, *p* = 0.017, Cohen’s *d* = 0.58, 95% CI for the mean difference [1.50, 13.59]). In summary, walking direction of peripheral BM stimuli played a role in attenuating the orienting of attention exerted by central arrow cues. This interference effect of BM cues may be rooted in the fact that social attention is more reflexive and temporally stable, whereas nonsocial attention gradually decreases and requires more top-down cognitive control to maintain (Liu et al., 2021).

### Experiment 3

Experiment 3 introduced peripheral arrow cues with a continuous increase in angles at the arrowhead and arrowtail to better match the dynamic BM cues, thereby eliminating the possibility that disparities in dynamic properties of stimuli might account for the observed asymmetric interference effect. Additionally, the size of BM cues was reduced to minimize potential confounds. Consistent with the findings of Experiment 1, the identical 2 × 2 repeated measures ANOVA only found a significant main effect of cue validity (*F*(1, 19) = 18.83, *p* < 0.001, *η*_*p*_^2^ = 0.50), neither the main effect of direction congruency (*F*(1, 19) = 0.16, *p* = 0.695, *η*_*p*_^2^ = 0.01) nor their interaction (*F*(1, 19) = 2.41, *p* = 0.137, *η*_*p*_^2^ = 0.11) reached significance (see Fig. 3E). Regardless of direction congruency between the peripheral and central cues, a significant BM-induced attentional orienting was revealed (valid, *M* ± *SE* = 350.42 ± 8.03 ms; invalid, *M* ± *SE* = 358.44 ± 7.83 ms). Normalized cueing effects provided additional evidence that there was no significant difference between the congruent and incongruent conditions (0.014 vs. 0.009, *t*(19) = 1.46, *p* = 0.161, Cohen’s *d* = 0.33, 95% CI for the mean difference [− 0.002, 0.012]). This indifference was also confirmed across all time bins (see Fig. 3F), as well as in the early and late periods (bin 1–80: *M* ± *SE* = 3.03 ± 2.78 ms, *t*(19) = 1.09, *p* = 0.289, Cohen’s *d* = 0.24, 95% CI for the mean difference [− 2.79, 8.86]); bin 81–160: *M* ± *SE* = 5.36 ± 3.43 ms, *t*(19) = 1.56, *p* = 0.135, Cohen’s *d* = 0.35, 95% CI for the mean difference [− 1.82, 12.54]) of the temporal analyses. In conclusion, Experiment 3 essentially replicated the results of Experiment 1, thus clearly demonstrating the robust resistance of BM-induced attention to interference from conflicting peripheral arrows.

Combined with Experiments 1, 2, and 3, an asymmetric interference effect was found when the direction of social cues (i.e., BM) conflicted with nonsocial cues (i.e., arrow). BM stimuli as peripheral cues could strongly affect the attentional processing of central arrow cues, but as central cues are immune to peripheral oppositely oriented arrows. This asymmetric interference effect, potentially reflecting susceptibility of central arrows to conflict, robust potency of peripheral BM in guiding attention, or their combination, suggests that BM cues hold attentional priority over arrow cues and might be coded by a specialized neural network (Troje & Westhoff, 2006). These results further supported the hypothesis that processing mechanisms of social attention might be dissociated from those of nonsocial attention.

### Experiment 4

**Fig. 4:**
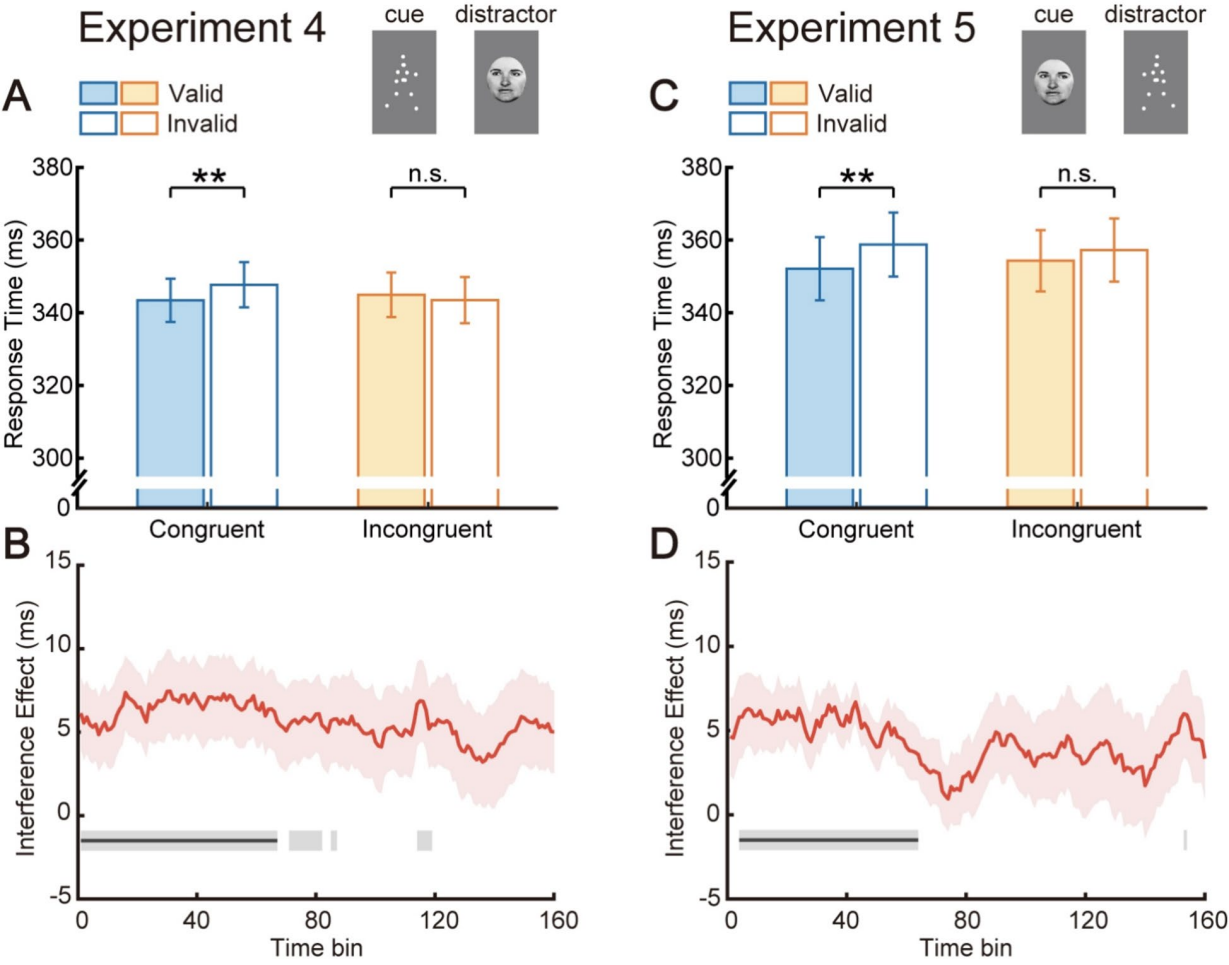
Results from Experiments 4–5. Whether adopting central BM versus peripheral eye gaze (Experiment 4, **A**, **B**) or central eye gaze versus peripheral BM (Experiment 5, **C**, **D**), the interference from peripheral social cues on attentional orienting evoked by central social cues was evident. **p < 0.01; n.s., not significant. In the panels below (**B**, **D**), red shaded areas represent standard errors; horizontal gray lines indicate time bins with significant interference effects at *p* < 0.05, while black lines indicate significant time bins after cluster-based permutation correction

Our daily life is crowded with diverse directional cues, and apart from conflicts between BM and nonsocial cues, BM also competes with social cues for limited attentional resources. Therefore, Experiment 4 additionally explored the interference effect of other social cues (i.e., eye gaze) on the attentional orienting evoked by BM cues. No significant main effects were found for cue validity (*F*(1, 19) = 0.98, *p* = 0.335, *η*_*p*_^2^ = 0.05) and direction congruency (*F*(1, 19) = 2.29, *p* = 0.146, *η*_*p*_^2^ = 0.11), while their interaction was significant (*F*(1, 19) = 8.66, *p* = 0.008, *η*_*p*_^2^ = 0.31). Reflexive attentional orienting stably existed when the orientation of central BM and peripheral gaze were aligned (343.43 ms vs. 347.69 ms, *t*(19) = 3.08, *p* = 0.006, Cohen’s *d* = 0.69, 95% CI for the mean difference [1.36, 7.18]), but disappeared when they were unaligned (344.92 ms vs. 343.49 ms, *t*(19) = − 0.71, *p* = 0.486, Cohen’s *d* = − 0.16, 95% CI for the mean difference [− 5.65, 2.79]; see Fig. 4A). In addition, the normalized cueing effect was also significantly smaller in the incongruent condition than that in the congruent condition (− 0.002 vs. 0.006, *t*(19) = − 3.12, *p* = 0.006, Cohen’s *d* = 0.70, 95% CI for the mean difference [− 0.014, − 0.003]). Consequently, these results collectively suggested that the reflexive attentional orienting triggered by BM cues was attenuated when the direction of peripheral gaze cues conflicted with BM cues.

Contrary to Experiment 2, the temporal trend of the interference effect indicated that time bins with significant cueing effects differences were bin 1–67 (see Fig. 4B). Moreover, the interference effect was significant in the first half of time bins (bin 1–80: *M* ± *SE* = 6.36 ± 2.17 ms, *t*(19) = 2.93, *p* = 0.009, Cohen’s *d* = 0.66, 95% CI for the mean difference [1.81, 10.90]), marginally significant in the second half (bin 81–160: *M* ± *SE* = 5.06 ± 2.60 ms, *t*(19) = 1.95, *p* = 0.067, Cohen’s *d* = 0.44, 95% CI for the mean difference [− 0.38, 10.50]). Thus, conflicting peripheral gaze cues could impact the BM-induced reflexive attentional orienting, thereby providing evidence that gaze cues, as another type of social cue, similarly engage a reflexive attentional process.

Collectively, when social cue BM was used as a central cue to steadily orient participants’ attention, the nonsocial cue in Experiments 1 and 3 (i.e., arrow) continuously failed to interfere throughout the task, whereas the other social cue in Experiment 4 (i.e., eye gaze) was able to generate significant interference. This asymmetric interference effect in Experiments 1, 3, and 4 again implied that there might exist a specialized brain mechanism for social but not nonsocial attentional orienting.

### Experiment 5

To investigate whether the interference effect between social stimuli obtained in Experiment 4 could also be found when their roles were switched, Experiment 5 was conducted. The 2 × 2 ANOVA revealed a significant main effect of cue validity (*F*(1, 19) = 9.39, *p* = 0.006, *η*_*p*_^2^ = 0.33), while no significant main effect of direction congruency (*F*(1, 19) = 0.11, *p* = 0.748, *η*_*p*_^2^ = 0.01). It is noteworthy that, similar to Experiment 4, the two-way interaction again reached significance (*F*(1, 19) = 7.38, *p* = 0.014, *η*_*p*_^2^ = 0.28). Consistent with findings of previous single-cue presentation studies (Friesen & Kingstone, 1998), central eye gaze cues induced a significant attentional cueing effect in the congruent condition (352.12 ms vs. 358.79 ms, *t*(19) = 3.53, *p* = 0.002, Cohen’s *d* = 0.79, 95% CI for the mean difference [2.72, 10.62]). In the incongruent condition, however, no significant cueing effect was found (354.33 ms vs. 357.24 ms, *t*(19) = 1.93, *p* = 0.069, Cohen’s *d* = 0.43, 95% CI for the mean difference [− 0.25, 6.08]; see Fig. 4C). Meanwhile, the difference between normalized cueing effects in the congruent and incongruent conditions was also significant (0.009 vs. 0.004, *t*(19) = 2.80, *p* = 0.011, Cohen’s *d* = 0.63, 95% CI for the mean difference [0.001, 0.009]). In sum, peripheral BM cues could affect the reflexive cueing effect evoked by central eye gaze cues.

Furthermore, the temporal results from Experiment 5 closely mirrored those observed in Experiment 4, with the interference effect significant in bin 4–64 (see Fig. 4D) and in the early period (bin 1–80: *M* ± *SE* = 4.68 ± 1.50 ms, *t*(19) = 3.13, *p* = 0.006, Cohen’s *d* = 0.70, 95% CI for the mean difference [1.55, 7.81]; bin 81–160: *M* ± *SE* = 3.76 ± 2.64 ms, *t*(19) = 1.42, *p* = 0.171, Cohen’s *d* = 0.32, 95% CI for the mean difference [− 1.77, 9.29]). In conclusion, the reflexive attentional orienting of central eye gaze cues could be affected by conflicts from peripheral BM cues.

Taken together, these converging findings of Experiments 4 and 5 confirmed a cross-category symmetric interference effect between eye gaze and BM, further suggesting that social attention induced by different types of social cues might involve shared neural mechanisms. Notably, this contrasts with the asymmetric interference between BM and arrows in Experiments 1–3, indicating two relatively distinct processes for social and nonsocial attention.

### Experiment 6

**Fig. 5:**
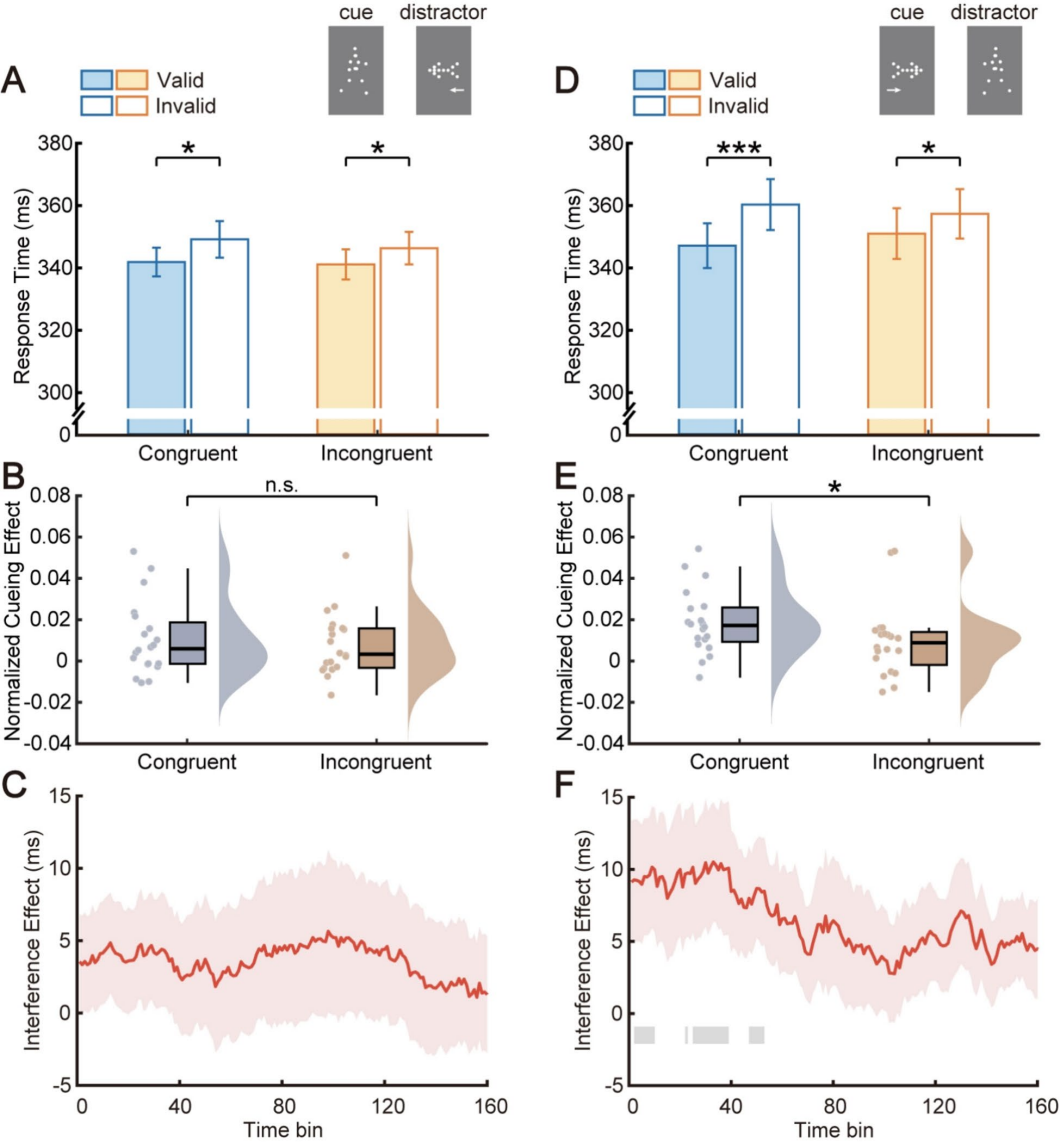
Results from Experiment 6. With improved stimulus matching and a within-subjects design, results of Experiment 6 replicated the asymmetric interference effects observed in Experiments 1–3. Attentional orienting elicited by BM cues robustly resisted interference from peripheral arrows (**A**, **B**, **C**) while reliably interfering with arrow-induced attention (**D**, **E**, **F**). ***p < 0.001; *p < 0.05. In the panels below (**C**, **F**), red shaded areas represent standard errors; horizontal gray lines indicate time bins with significant interference effects at *p* < 0.05, while black lines indicate significant time bins after cluster-based permutation correction

To validate the asymmetric interference effects between BM and arrow cues, Experiment 6 employed a within-subjects design with dynamically enhanced arrow stimuli that incorporated implied motion to better match BM properties. This approach minimized between-subject variability (Montoya, 2023) and assessed whether the asymmetry reflects differences between social and nonsocial attention mechanisms. When central BM cues were presented with peripheral arrow cues, results were in line with those of Experiments 1 and 3. The identical 2 × 2 ANOVA only revealed a significant main effect of cue validity (*F*(1, 19) = 7.48, *p* = 0.013, *η*_*p*_^2^ = 0.28), with no significant main effect of direction congruency (*F*(1, 19) = 3.12, *p* = 0.094, *η*_*p*_^2^ = 0.14) or their interaction (*F*(1, 19) = 0.55, *p* = 0.467, *η*_*p*_^2^ = 0.03). BM cues elicited robust attentional cueing effects irrespective of directional congruency with peripheral arrow cues (valid, *M* ± *SE* = 341.53 ± 4.67 ms; invalid, *M* ± *SE* = 347.74 ± 5.48 ms; see Fig. 5A). The reflexivity of BM-induced attentional orienting was further corroborated by equivalent normalized cueing effects between congruent and incongruent conditions (0.010 vs. 0.007, *t*(19) = 0.67, *p* = 0.513, Cohen’s *d* = 0.15, 95% CI for the mean difference [− 0.006, 0.011]; see Fig. 5B). Consistently, no significant interference effect was observed across all time bins (see Fig. 5C), including both early and late task period (bin 1–80: *M* ± *SE* = 3.60 ± 4.01 ms, *t*(19) = 0.90, *p* = 0.381, Cohen’s *d* = 0.20, 95% CI for the mean difference [− 4.80, 11.99]; bin 81–160: *M* ± *SE* = 3.56 ± 4.60 ms, *t*(19) = 0.78, *p* = 0.448, Cohen’s *d* = 0.17, 95% CI for the mean difference [− 6.06, 13.19]). To sum up, Experiment 6 replicated the findings of Experiments 1 and 3, reaffirming the automaticity and reflexivity of BM in guiding attention against interference from peripheral arrows throughout the task.

On the contrary, when arrow cues were presented centrally and BM cues peripherally, ANOVA revealed a significant cue validity × direction congruency interaction (*F*(1, 19) = 4.96, *p* = 0.038, *η*_*p*_^2^ = 0.21), with a main effect of cue validity (*F*(1, 19) = 21.40, *p* < 0.001, *η*_*p*_^2^ = 0.53) but not direction congruency (*F*(1, 19) = 0.09, *p* = 0.766, *η*_*p*_^2^ < 0.01), implying a modulation of directional congruency on the magnitude of arrow-induced attention. Post hoc *t* tests found that arrow cues induced significant cueing effects in both congruent (347.13 ms vs. 360.33 ms, *t*(19) = 5.13, *p* < 0.001, Cohen’s *d* = 1.15, 95% CI for the mean difference [7.81, 18.57]) and incongruent (351.00 ms vs. 357.34 ms, *t*(19) = 2.39, *p* = 0.028, Cohen’s *d* = 0.53, 95% CI for the mean difference [0.78, 11.89]; see Fig. 5D) conditions. Critically, this modulation from peripheral BM cues was further confirmed by normalized cueing effects, which differed significantly between congruent and incongruent conditions (0.018 vs. 0.009, *t*(19) = 2.14, *p* = 0.046, Cohen’s *d* = 0.48, 95% CI for the mean difference [0.0002, 0.018]; see Fig. 5E). Although single time bins did not reveal significant interference effects after correction, a significant interference effect emerged when averaged across the early period (bin 1–80: *M* ± *SE* = 8.11 ± 3.82 ms, *t*(19) = 2.12, *p* = 0.047, Cohen’s *d* = 0.47, 95% CI for the mean difference [0.12, 16.11]; bin 81–160: *M* ± *SE* = 4.80 ± 3.10 ms, *t*(19) = 1.55, *p* = 0.138, Cohen’s *d* = 0.35, 95% CI for the mean difference [− 1.68, 11.28]; see Fig. 5F). In summary, these findings collectively verify that peripheral BM cues could interfere with arrow-induced attention.

Across all experiments, BM and arrow stimuli exhibited an asymmetric interference effect favoring BM signals (Experiments 1–3 and 6), while BM and eye gaze stimuli possessed a symmetric mutual interference effect (Experiments 4–5). This striking dissociation, which may stem from the vulnerability of central arrows to conflict, the superior attentional potency of peripheral social cues, or both factors, supports specialized neural networks for social attention that are distinct from, yet more reflexive than, nonsocial attention.

## Discussion

Previous studies in the field of BM-induced attention have traditionally been limited to isolated cues, whereas in the real world, BM cues are always embedded in complex environments and need to compete for attention. The current study extends the existing literature by innovatively exploring how attentional systems resolve potential conflicts arising from simultaneously perceived BM and other types of cues. Our results showed that cueing effects induced by central BM cues exhibited stable resistance to interference from peripheral arrow cues for a relatively long time, while cueing effects induced by central arrow cues disappeared under peripheral BM disturbances. This asymmetric interference effect strongly highlights the automatic extraction and reflexive advantage of walking direction of BM over arrow cues, triggering attentional orienting even amidst stimuli indicating opposite locations. Conversely, a cross-category and symmetric interference effect was observed between BM and eye gaze cues, suggesting that social cues induce attentional shifts with comparable reflexivity. From a real-world multi-stimulus conflicting perspective, these findings provide new compelling evidence for a unique processing mechanism underlying social attention induced by different social cues, which may not apply to nonsocial attention. This mechanistic insight underscores the crucial role of social signals as potent attentional cues in domains where rapid, accurate attentional guidance amid conflict is critical.

The attentional orienting triggered by walking direction of BM has been proven to be reflexive in single-cue presentations, independent of observers’ awareness of its biological nature (Wang et al., 2014) and resistant to temporal decay as task proceeds (Liu et al., 2021). In our study, even with an increased trial number (from 16–30 to 80 trials per condition) and nonpredictive stimuli (i.e., central and peripheral cues) consistent with established interference paradigms (Besner et al., 2021a; Nummenmaa & Hietanen, 2009), cueing effects of BM remained stably immune to peripheral opposite arrows while strongly disturbing central arrow processing. Critically, increased trial number enabled a sliding window analysis (Esterman et al., 2013; Liu et al., 2021), which allowed for a more fine-grained temporal analysis compared to previous coarse divisions (e.g., early versus late halves (van Laarhoven et al., 2018); experimental blocks (Lin et al., 2016; Shalev et al., 2016)). This approach provided definitive evidence that BM-induced attention was robust enough to withstand arrow interference throughout the entire task. This result well fulfills the criterion for automatic processes, which stipulates that an automatic process can interfere with other processes but is unaffected by them (Besner et al., 2021b). Therefore, our study unambiguously verifies the reflexive nature of BM-induced attention, which is potentially rooted in its evolutionary advantage over symbolic cues (Lu et al., 2024). Among social organisms, rapid perception and interpretation of other creatures’ focuses is essential for survival, offering clues for food acquisition (Galef & Giraldeau, 2001), predator avoidance (Griffin, 2004), and cooperation seeking (Chevallier et al., 2012). Given these life-or-death outcomes, spontaneous attentional shifts to BM signals emerge early in human development (Bardi et al., 2015; Lunghi et al., 2019, 2020) and are also observed in nonhuman species. For instance, newly hatched chicks, despite lacking visual experience, aligned their bodies with walking direction conveyed by point-like animations (Vallortigara & Regolin, 2006). As a result, when presented with biologically meaningless arrow cues, even nonpredictive BM may be prioritized in competition for attentional resources and unilaterally interfere with response to arrows. By displaying different cues simultaneously, our study proposes a more stringent criterion for reflexive attentional orienting, namely being shielded from interference from conflicting peripheral cues, which better matches real-world complexity where attention must operate amid competing cues.

Furthermore, the asymmetric interference effect between BM and arrow cues suggests that, from a conflict-resistance perspective, arrow-induced attention is less reflexive and more susceptible to top-down cognitive control (Friesen et al., 2004; Liu et al., 2021; Ristic et al., 2007; Schmitz et al., 2024). Although nonsocial cues such as arrows can elicit attentional shifts even when they are target-uninformative (Hommel et al., 2001; Tipples, 2002), findings predominantly rely on single-cue presentations. However, given that real life involves multiple cues and the inevitable resultant conflicts, a stricter criterion is needed to reassess the reflexivity of nonsocial attention. Importantly, our study provides a more direct comparison of attentional orienting induced by BM and arrow cues, revealing a unique mechanism underlying BM processing that is impervious to interference from nonsocial cues. The distinction between BM- and arrow-mediated attention may arise because arrows are overlearned symbols with flexible directional interpretations (Ristic & Kingstone, 2012; Schmitz et al., 2024). Not surprisingly, attentional shifts to such nonsocial cues lack an evolutionary basis and are primarily shaped by environmental factors (Wang et al., 2020). Therefore, arrow cues failed to interfere with BM cues and were strongly affected by them when guiding spatial orientation in conflict.

It is noteworthy that, in contrast to asymmetric interference effects between social and nonsocial cues, interference effects between different social cues are symmetric. Specifically, when directions of BM and eye gaze conflicted, peripheral gaze cues reduced BM-induced attention. Similar results were observed when the two cues switched positions. These findings indicate that orienting shifts by social cues show an equivalent level of reflexivity, an idea that is also validated by studies separately comparing single cues (Driver et al., 1999; Friesen & Kingstone, 1998; Liu et al., 2021; Shi et al., 2010). Similar to BM, even presented as irrelevant peripheral cues, nonpredictive gaze cues are hard to inhibit and indeed trigger attentional shifts. In addition, this cross-category and symmetric interference effect further indicates that directional information from simultaneously perceived social cues is voluntarily integrated for attentional orienting (Langton, 2000), implying the existence of a general underlying mechanism shared by different types of social attention behaviors. This assertion is supported by the cross-category adaptation aftereffects (Ji et al., 2020) and reliable common genetic effects (Wang et al., 2020) between social attention evoked by BM and eye gaze. Crucially, neuroimaging evidence has demonstrated that the superior temporal sulcus (STS) region could decode attentional orienting across BM and gaze cues (Wang et al., 2024). Based on these findings, our study is the first to directly compare BM and gaze cues in a single trial. The symmetric and rapid interference effect suggests, within the dimension of conflict, a shared mechanism for reflexive social attention induced by different social cues, providing strong evidence for the “social attention detector” (Ji et al., 2020; Wang et al., 2020).

Altogether, our dual-cue conflict paradigm revealed that attentional orienting to social cues is more reflexive than that to nonsocial cues in conflict situations, providing compelling evidence for the distinctiveness of social attention. Previous studies comparing social and nonsocial attention predominantly rely on isolated displays of gaze and arrow cues, reporting comparable cueing effects (Wang et al., 2024) but considerable discrepancies regarding their functional properties and neural substrates. In addition to behavioral findings that social attention is more reflexive (Friesen et al., 2004; Liu et al., 2021; Ristic et al., 2007), some neuroimaging studies indicate that gaze and arrows recruit distinct attention systems: the dorsal attentional network for arrow cueing and the ventral attention network for gaze cueing (Callejas et al., 2014; Joseph et al., 2015). In contrast, other studies have reported no discernible difference between these two types of attentional orienting, either in terms of behavioral effects (Chacón-Candia et al., 2023; Singh et al., 2022; Tipples, 2008) or neural activation (Callejas et al., 2014; Greene et al., 2009; Sato et al., 2009; Tipper et al., 2008; Uono et al., 2014). Such discrepancies may stem from limited sensitivity of classical cueing paradigms to capture distinctive characteristics that differentiate gaze from arrow cues (Birmingham & Kingstone, 2009), as cues are primarily compared along their shared dimension of directional information (Gibson & Kingstone, 2006). Here, employing BM cues and competing displays helps to resolve the long-standing discrepancies in previous single-cue studies. Our noteworthy findings disclosed the reflexive superiority of social cues over nonsocial cues in guiding attention, aligning with a wealth of studies that underscore the role of social but not nonsocial cues in higher cognitive processes (e.g., mentalizing, working memory, and time perception; Ge et al., 2024; Huang et al., 2024; Marotta et al., 2012; Yu et al., 2023; Yuan et al., 2023; Zhang et al., 2025). Furthermore, this reflexive dominance suggests that an underlying neural architecture is specialized for involuntary social attention, a notion substantiated by a recent fMRI study identifying the right STS and superior temporal gyrus (STG) as regions capable of decoding attentional orienting across social (eye gaze and BM) but not nonsocial (arrow) cues (Wang et al., 2024). Collectively, by introducing conflict as a critical dimension for evaluating attentional reflexivity, the current study offers novel insights into the distinctive nature of social attention.

The identified reflexive priority of social signals in conflict carries immediate translational value across diverse applied domains. First, this priority can be leveraged to optimize navigation and public signaling systems. For instance, dynamic human-figure indicators may outperform conventional arrow-based signage in transportation hubs, commercial centers, or healthcare facilities, since social signals could function as robust attentional anchors amidst visual clutter or high cognitive demand. Such advantage becomes particularly critical where attention-driven decision-making is paramount to safety (e.g., high-speed driving, incident response, or emergency evacuations; Hinton et al., 2024; Sundfør et al., 2019; van Harten et al., 2021). Beyond physical environments, these principles extend to digital interface design across 2D platforms (e.g., websites, mobile applications, and information dashboards) and 3D spaces (e.g., virtual or augmented reality deployed in navigation, industrial operations, or surgical guidance), where embedding subtle social cues can effectively guide user’s focus toward critical information (Appel et al., 2012; Bönsch et al., 2024). Particularly in emergency alert systems and safety–critical notifications, embedding social signals (a sudden BM cue within an arrow‑based navigation display) may redirect attention to imminent hazards, ensuring vital warnings cut through information-rich interfaces.

It should be noted that the current samples included only healthy adults; however, not all individuals exhibit equal adeptness in following others’ walking direction. Impaired social orienting has been well-documented in individuals with autism spectrum disorder (ASD; American Psychiatric Association, 2022), serving as a core clinical indicator for early diagnosis (Dawson et al., 2012). Despite this, some laboratory experimental studies have reported relatively intact attentional shifts induced by social cues (e.g., eye gaze) in ASD (Kirchgessner et al., 2015; Pruett et al., 2011; Rombough & Iarocci, 2013); but see (Gillespie-Lynch et al., 2013; Goldberg et al., 2008; Yang et al., 2024; Zhao et al., 2017). These studies primarily tested social attention through simple single-cue displays, and individuals with ASD beyond school age may vicariously utilize low-level directional features or nonsocial mechanisms to fulfill tasks (Nation & Penny, 2008; Wang et al., 2020; Yang et al., 2024). Given the complexity of the real world, it is probable that individuals with ASD consistently show diminished social attention when processing conflicting directional cues (Dichter & Belger, 2007; Rombough & Iarocci, 2013; Zhao et al., 2017). Therefore, our conflict paradigm may offer a more reliable behavioral marker for ASD, providing a sensitive and ecologically valid tool that could enhance early screening and inform interventions aimed at improving social attention ability in naturalistic settings.

Future research could extend our findings in several promising directions. Our screen-based paradigm prioritized perceptual discriminability and ensured comparable attentional effects across cue types (Wang et al., 2024), yet this necessarily constrained stimulus matching (e.g., size, dynamics, information richness) and ecological realism. Emerging virtual reality (VR) and augmented reality (AR) technologies offer transformative opportunities to transcend these constraints (Castellana et al., 2025; Haskins et al., 2022), enabling naturalistic presentation of diverse social and nonsocial signals while maintaining experimental control. It is also worth noting that, although point-light BM displays eliminate discernible facial features and explicit gaze cues, observers may automatically extract facing direction or infer gaze direction and accordingly orient their attention. Future studies could address this interpretive limitation by employing local BM without body configuration (Lu et al., 2024) or backward-walking BM stimuli (Ding et al., 2017), thereby isolating the contribution of pure kinematic signals from implied directional information. VR and AR platforms further permit orthogonal manipulation of agents’ gaze and walking direction (e.g., gaze aligned with, away from, or opposite to walking direction) to disentangle their independent and interactive effects on attentional orienting. Additionally, eye-tracking methodologies could validate the covert nature of social attention observed here or, shed light on how attention is dynamically allocated amid conflict in paradigms permitting eye movement. As such, integrating these technologies with the conflict paradigm holds the potential to illuminate how reflexive social attention identified in the laboratory unfolds in our daily life.

In conclusion, the current study reveals that walking direction of BM can reflexively trigger attentional orienting even in the presence of conflicting peripheral arrow cues and can further interfere with the arrow processing. Furthermore, social cues (i.e., BM, eye gaze) exhibit a cross-category and symmetric interference effect. These findings, in the context of conflict mirroring real-life situations, provide novel evidence that social attention induced by various social cues shares a specialized mechanism potentially distinct from nonsocial attention, with broad application ranging from optimizing safety–critical signaling and human–machine interfaces to advancing early diagnosis and intervention for ASD.

## Data Availability

We meticulously detail our methodology, including the sample size, data exclusion, manipulations, and measures. Statistical analyses were conducted using JASP Version 0.19.1 and G*Power Version 3.1. While this study was not pre-registered, full transparency is maintained through open data and analysis code accessible at Science Data Bank (10.57760/sciencedb.18066). Materials used in our research are widely available.
